# Cross-Device Computation Coordination for Mobile Collocated Interactions with Wearables

**DOI:** 10.3390/s19040796

**Published:** 2019-02-15

**Authors:** Hyoseok Yoon, Choonsung Shin

**Affiliations:** Korea Electronics Technology Institute, Mapo-gu, Seoul 03924, Korea

**Keywords:** context-awareness, cross-device, Internet-of-Things, computation offloading, machine learning, mobile interaction, wearables

## Abstract

Mobile devices, wearables and Internet-of-Things are crammed into smaller form factors and batteries, yet they encounter demanding applications such as big data analysis, data mining, machine learning, augmented reality and virtual reality. To meet such high demands in the multi-device ecology, multiple devices should communicate collectively to share computation burdens and stay energy-efficient. In this paper, we present a cross-device computation coordination method for scenarios of mobile collocated interactions with wearables. We formally define a cross-device computation coordination problem and propose a method for solving this problem. Lastly, we demonstrate the feasibility of our approach through experiments and exemplar cases using 12 commercial Android devices with varying computation capabilities.

## 1. Introduction

Recent rapid developments in low-power microcontrollers, application processors, wireless chips and sensors are paving the way for consumer-level Internet-of-Things (IoT), mobile devices and wearables. At this rate, integrations of IoT in small, mobile and wearable form factors with even smaller batteries will be constantly and ubiquitously emerging. On one hand, IoTs are increasingly required to be stand-alone, always-on and long-lasting to continuously monitor their surroundings. On the other hand, IoTs are encountering practical challenges to perform demanding algorithms for big data analysis, data mining, machine learning, augmented reality (AR) and virtual reality (VR). In this regard, researchers are actively exploring a means of strategically and efficiently managing constrained resources of IoT such as its processing power and limited batteries. Through emerging and enabling technologies in edge computing [1,2], fog computing [3] and cross-device interaction (XDI) [4,5], various common tasks in homes and work places can be performed in coordination of multiple devices. To provide such a solution, we need to accurately detect and assess IoTs for their capabilities as well as their deficiencies. Moreover, we need to employ a logical process to help different IoTs co-operate. Ideally, we dream of context-aware IoTs that communicate in a way to collectively share computation burdens and stay energy-efficient.

In this paper, we present a cross-device computation coordination (XDCC) method for scenarios of mobile collocated interactions with wearables and IoT. We first introduce relevant concepts and preliminaries to define what a computation coordination problem is. Then, we propose a method and its components to support and solve the defined problem systematically. Lastly, we demonstrate general needs and feasibility of using our proposed method for computation coordination through several experiments and exemplar cases. For this purpose, we use commercial Android smartphones and Wear OS smartwatches of varying computation capabilities, to represent advanced mobile and wearable IoTs. Our contribution in this paper is as follows.

1. **Definition of the Cross-Device Computation Coordination Problem.** We define a cross-device computation coordination (XDCC) problem considering on-device static and dynamic characteristics of IoTs, mobile devices and wearables.
2. **Design and Implementation of Cross-Device Computation Coordination Method.** We design an XDCC method consists of contextual device profiling, task time measurement and side information lookup.
3. **Benchmark Scores as Usable Context and Side Information.** We propose a concept of using third-party benchmark scores to characterize relative performance of IoT, mobile devices and wearables, which can be used as side information for our XDCC method.
4. **Experiments and Exemplar Cases on 12 Commercial Devices.** We demonstrate and evaluate our proposed XDCC method on eight Android smartphones and four Wear OS smartwatches through several experiments and exemplar cases.

## 2. Overview

In this section, we introduce background concepts and preliminaries followed by a formal definition of a cross-device computation coordination problem.

### 2.1. Concepts

We first introduce two important concepts that are frequently referenced in this paper.

**Figure 1:**
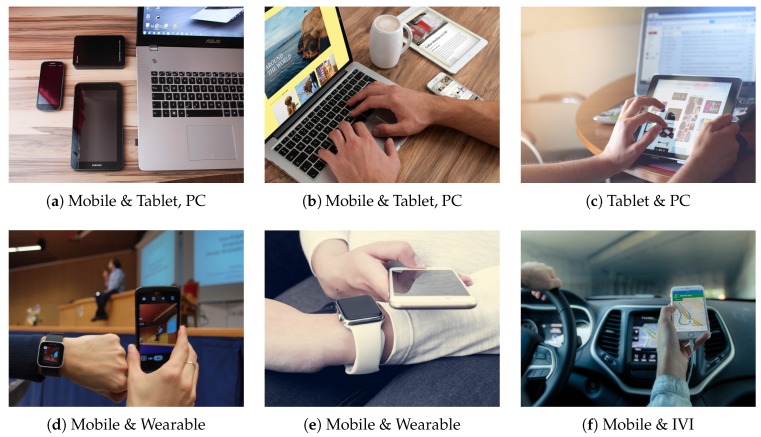
MCI examples with mobile, tablet, PC, wearables and IVI.

**Concept 1 (Mobile Collocated Interactions).** Mobile collocated interactions (MCI) is a concept to describe various *situations of users (or a user)* participating in collaborative activities with multiple devices simultaneously or in turn [6,7]. Often, these multiple devices collocate in the user’s vicinity and include a personal mobile device. Figure 1 depicts typical MCI examples we encounter everyday. Figure 1a–c all demonstrate a common office or home environment where the user has access to multiple devices including a smartphone, tablet and PC. Figure 1d,e illustrate MCI with wearables where the user is equipped with a smartwatch. Figure 1f shows MCI with an in-vehicle infotainment (IVI) to share information between the IVI and the user’s smartphone. A *specific type of user interaction* with such multi-devices in MCI is known as cross-device interaction (XDI) [4,5].

For example, real use cases of XDI explored in recent studies include using a wearable device (i.e., the Google Glass) with smartphones to offload real-time, computationally heavy and energy-consuming processes such as text recognition, face detection, marker tracking and gesture recognition [8]. Moreover, XDI applications contribute to various application domains covering knowledge work, home computing, data exploration, mobile computing, games/installations, collaboration, education, health and software development as identified by Brudy et al.’s cross-device taxonomy [5].

**Figure 2:**
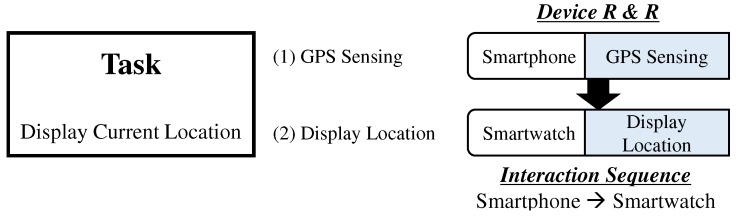
A breakdown of an exemplar XDCC.

**Concept 2 (Cross-Device Computation Coordination).** We define a concept of cross-device computation coordination (XDCC) as a means of sharing and integrating information and resources between the connected devices in MCI so that each device can accomplish their part in support of a mutual objective of completing a task. Most suitable target tasks for XDCC are tasks that require real-time, computationally heavy and energy-consuming processes. A typical example includes machine learning applications (i.e., text recognition, face detection, marker tracking and gesture recognition) and sensor-based data processing. For example, to display current location on a smartwatch application, GPS signals received in a smartphone can be shared with a paired smartwatch. To perform XDCC, we need to specify roles and responsibilities (R & R) of each device as well as a particular interaction sequence between devices as illustrated in Figure 2.

### 2.2. Preliminary

We introduce basic definitions that are used throughout this paper.

**Definition 1 (Device Resources).** A set of *n* resource items, $resources=\{r_{1},\cdots ,r_{n}\}$, represents a device’s resources where a resource item may be an on-device sensor (i.e., accelerometer, light sensor, proximity sensor) or a hardware component (i.e., display, speaker).

**Definition 2 (Device Profile).** A device profile $D_{i}$ for a task consists of available resources of the device, an *optional* time spent to perform the task where *i* is a unique identifier for the device and side information attributes, $D_{i}=\left(resources,time\_s,side\_info\right)$. $D_{i\cdot resources}$ indicates a set of available resources on the device. $D_{i\cdot time\_s}$ represents on-device completion time for executing the specified task. $D_{i\cdot side\_info}$ indicates side information such as the device’s benchmark score.

**Definition 3 (Task Description).** A task description *T* is represented with resources and a time limit attribute as T=(resources,limit). $T_{\cdot resources}$ indicates a set of *k* resource items required for completing the task, represented by $T_{\cdot resources}=\left\{r_{1},\cdots ,r_{k}\right\}$. $T_{\cdot limit}$ indicates the time limit for completing the task in milliseconds. For example $T_{\cdot limit}=500$ indicates that the task should be completed within 500 ms.

### 2.3. Problem Definition

With previously defined concepts and preliminaries, we formally define XDCC as a problem of finding possible XDCC policies.

**Definition 4 (Cross-Device Computation Coordination Policy).** Given two device profiles ($D_{1}$ and $D_{2}$) and a task description *T*, an XDCC policy $XD_{policy}$ is an implementation of a teamwork sequence between two paired devices, $XD_{policy}=\langle D_{1},D_{2}\rangle$, where $D_{1}$ specifies a main device that first performs time-critical computational part of the task followed by a supporting device $D_{2}$ performing the rest of the task. More precisely $XD_{policy}$ is valid if $D_{1}$ satisfies two conditions (i.e., contains all the required resources and conforms to the time-limit) of performing the task. Note that $XD_{policy}=\langle D_{1},D_{2}\rangle$ and $XD_{policy}=\langle D_{2},D_{1}\rangle$ are two different XDCC policies.

### 2.4. Method Overview

**Figure 3:**
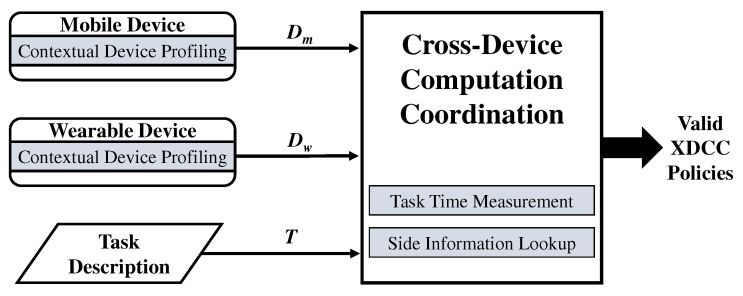
Method overview.

Figure 3 outlines the overview of our proposed method. Our method structure is twofold. First, multi-devices for XDI such as mobile devices, wearables and IoTs in MCI individually produce their device profiles. Second, using device profiles and task description, we explore possible solutions for XDCC policies in either one of the profiled devices or partially on another third-party central server. In the proposed method, the following three procedures are performed.

- Step 1—*Contextual Device Profiling*, which scans multi-devices for available on-device resources which include sensors and hardware components. The output of this step is a device profile for each device (cf. Section 3.2).
- Step 2—*Task Time Measurement*, which performs a task or a simulation of the task to measure the time for completing the task on the device. The output of this step is on-device task completion time (cf. Section 3.3).
- Step 3—*Side Information Lookup*, this optional step integrates external knowledge into the method when Step 2 is not viable. This step looks up previously recorded benchmark scores for references and outputs a pre-stored or an estimated task completion time (cf. Section 3.4).

## 3. Cross-Device Computation Coordination Method

In this section, we present our rationale for the XDCC problem and describe the three procedures of our XDCC method in detail.

### 3.1. A Rationale for XDCC in a Multi-Device Environment

Mobile devices, wearables and IoTs have different form factors, sizes and user interfaces to serve various use cases and applications. By using two or more devices simultaneously or in turn, we create new opportunities that benefit from the extended and combined resources of multi-devices. To do so, we compare devices to find a device that is both physically capable and computationally more efficient than its partner. There are two factors we consider for coordinating a computational task *T* in MCI between two devices $D_{1}$ and $D_{2}$. We describe our reasoning with simple examples for brevity.

1. **Physical Capability.** The first principle is related to the physical capabilities of the devices. Consider an MCI case where $D_{1}$ is physically capable to perform a task *T* while $D_{2}$ is not. Then *T* can be performed in only $D_{1}$, since $D_{2}$ is not compatible with *T* at all. For example, offloading *T* to $D_{1}$ is a rational decision to make when *T* requires a speaker and $D_{1}$ is the only device with a speaker.
2. **Computation Efficiency.** If *T* is physically feasible on both $D_{1}$ and $D_{2}$, then we should consider our second principle which is related to computation efficiency. If *T* on $D_{1}$ takes 10 s and *T* on $D_{2}$ takes 100 s, then it is a plausible decision to offload a time-critical task *T* to $D_{1}$ to save time. If *T* on $D_{1}$ is known to consume 5% of battery whereas *T* on $D_{2}$ is known to consume 20% of battery, then offloading to $D_{1}$ is a better choice to save scarce resource in the multi-device environment. Even though different priorities and strategies can be used, time and battery usages are two tightly coupled concerns for MCI with wearables and IoTs.

### 3.2. Contextual Device Profiling

The first procedure of the XDCC method is Contextual Device Profiling (CDP) that captures a snapshot of the devices. Various types of multi-devices in MCI (i.e., mobile devices, wearables and IoTs) can be described in static and dynamic context of each device. Static context of a device refers to characteristics of the device that persist in the lifespan of the device. An example is a device specification that describes hardware components (i.e., processors, screen size, battery capacity, weight) and presence of embedded sensors of the device (i.e., accelerometer and proximity sensor). Dynamic context of a device refers to attributes that change through user interactions with the device. Examples include temperature of the device, current battery level, load of a central processing unit (CPU) and a number of running applications. Many studies have extracted different contextual usages from personal mobile devices such as smartphones. Shin et al. derived contextual information from smartphone sensors and its usages such as GPS, time, battery, app, cellular network, setting, 3D accelerometer, illumination, screen, call-SMS, Wi-Fi and Bluetooth [9]. In another work, Shin et al. extracted usage features of smartphones from its general usage, battery usage, data usage, push event usage, touch inputs, session usage and app usage [10].

In our work, we take into account both static and dynamic context of cross-devices in MIC. We retrieve various on-device contextual information to capture a snapshot of the device’s capabilities and resources. To do so, we pay particular attention to the device’s communication, memory, battery and sensors. The device’s employed communication technology and its status are significant pieces of information, because they can result in a preferred transfer method to be used for implementing XDI. Similarly, current memory usage indicates unmet needs for future data or computation offload. Furthermore, battery shortages of a device impose physical and usability limitations on users. In this regard, reducing battery consumption and improving battery life are both academic and engineering research topics for ubiquitous computing [11] and IoT [12]. To understand a device’s performance, quantified metrics for a device such as CPU load percentage, numbers of running applications and processes, are relevant information to collect. However, recent versions of Android prevent access to /proc/stat for obtaining system and process related information due to security concerns (Android O prevents access to /proc/stat, https://issuetracker.google.com/issues/37140047). Therefore, such information is excluded in our suite of on-device sensing for CDP. Table 1 lists different categories of static and dynamic context for implementing our CDP.

We developed and implemented a suite of on-device context sensing to obtain static and dynamic context from the cross-devices. As described in Table 1, we analyze current statuses and usages of the devices in terms of memory, communication, benchmark scores, sensors and battery.

- **Device Name.** A device model (Build.MODEL) is used as a consumer friendly name or a unique ID for identifying the device.
- **Screen.** The screen resolution size in pixels is retrieved, which is important for personalizing or retargeting graphical user interfaces.
- **Memory.** The available memory on the system, the total memory accessible by the kernel and the threshold of available memory are collected by accessing ActivityManager.MemoryInfo (https://developer.android.com/reference/android/app/ActivityManager.MemoryInfo) in Android API to determine current memory usage and a low-memory situation.
- **Battery Status.** Battery charging status, remaining battery percentage and battery temperature information are reported using BatteryManager (https://developer.android.com/reference/android/os/BatteryManager).
- **Charging.** A source for charging the battery is identified (USB or AC).
- **Wi-Fi.** Current Wi-Fi network connection statuses (i.e., connected or not and signal strength if connected) are reported using ConnectivityManager (https://developer.android.com/reference/android/net/ConnectivityManager) of Android API.
- **Bluetooth.** A current Blutooth connection status (i.e., on or off) is reported using BluetoothAdapter (https://developer.android.com/reference/android/bluetooth/BluetoothAdapter) of Android API.
- **Sensors.** All available sensors on the device are retrieved using SensorManager (https://developer.android.com/reference/android/hardware/SensorManager).

### 3.3. Task Time Measurement

The second procedure of the XDCC method is Task Time Measurement (TTM) that measures the time a device takes to perform the task *T* (i.e., from start to finish). This measurement process can be actually performed on the device or simulated. Consider a task that classifies a stream of sensor data into one of pre-defined user gestures. To complete this task, a series of computation is performed. For example, the stream of sensor data is pre-processed, useful features are extracted and a candidate gesture that matches with those extracted features is returned as the output of the task. Note that we are measuring the time to complete the task. So the output of TTM is given in the unit of time which is different from the output of the task (i.e., a type of user gesture). In practice, devices in MCI have different hardware specifications (i.e., CPU, GPU and RAM). Consequently, their performance will vary even with the same task. In our proposed method, it is an engineer’s responsibility to develop and supply a module that correctly measures the task completion time. We developed Android and Wear OS-compatible TTM for our work. Algorithm 1 shows a pseudocode for CDP and TTM.

**Algorithm 1** An algorithm for computing XDCC policies**Input:** A task description *T*, a mobile device profile $D_{m}$, a wearable device profile $D_{w}$**Output:** A set of capable cross-device policy pairs $XD_{policy}$
 1:$XD_{policy}\leftarrow ⌀$, $D_{m}._{time\_s}\leftarrow \infty$, $D_{w}._{time\_s}\leftarrow \infty$ /* initialization */ 2:$D_{m}._{resources}\leftarrow$ContextualDeviceProfiling($D_{m}$) 3:**if**$\left(T._{resources}\in D_{m}._{resources}\right)$**then** /* check for mobile-centric interaction */ 4:    $D_{m}._{time\_s}\leftarrow$ TaskTimeMeasurement($D_{m}$) /* measure time to complete *T* on $D_{m}$ */ 5:    **if** $\left(D_{m}._{time\_s}\leq T._{limit}\right)$ **then** 6:        $D_{w}._{time\_s}\leftarrow 0$, $XD_{policy}\leftarrow \left(D_{m},D_{w}\right)$ /* add a new pair */ 7:    **end if** 8:
**end if**
 9:
**if** $\left(T._{resources}\in D_{w}._{resources}\right)$ **then**
10:    $D_{w}._{time\_s}\leftarrow$ TaskTimeMeasurement($D_{w}$) /* measure time to complete *T* on $D_{w}$ */11:    **if** $\left(D_{w}._{time\_s}\leq T._{limit}\right)$ **then** /* check for wearable-centric interaction */12:        **if** $D_{m}._{time\_s}=\infty$ **then**13:           $D_{m}._{time\_s}\leftarrow 0$14:        **end if**15:        $XD_{policy}\leftarrow \left(D_{w},D_{m}\right)$ /* add a new pair */16:    **end if**17:
**end if**
18:$XD_{policy}\leftarrow$SortByTaskTime($XD_{policy}$) /* sort pairs by task time of the first device */19:
**return** $XD_{policy}$



### 3.4. Side Information Lookup

The third procedure of the XDCC method is Side Information Lookup (SIL) which is an optional step that integrates external knowledge into our method. SIL is designed to replace repetitive TTM on devices with similar configuration. For example, performance of two devices with the same CPU model, the same amount of memory and the same amount of storage are closely comparable, as witnessed by several benchmark scores. Indeed, an accurate CDP provides useful information. However, to be used in an application running various computationally intensive tasks, a quantifiable and comparable metric is required. Therefore, we use two benchmarks (AnTuTu (http://www.antutu.com/en/index.htm, a commonly used software benchmarking tool for benchmarking smartphones) and VFP (https://dench.flatlib.jp/app/vfpbench, a benchmarking tool for VFP (Vector Floating Point) technology, which is the FPU (Floating-Point Unit) coprocessor extension to the ARM architecture)) to holistically quantify and capture relative “computational level” or “computational readiness” of each mobile/wearable/IoT devices. We leverage this by measuring device performance on various aspects, including CPU, GPU, UX, MEM and single, double precision floating point, single and multi-thread. We envision using a publicly open lookup table that contains device profiles, TTM per tasks and aforementioned benchmark scores. Using this lookup table, we implement SIL as a lookup function that outputs TTM. There are three SIL use cases with different inputs. Algorithm 2 shows a pseudocode for triggering different and hierarchical use cases in SIL.

1. SIL(DeviceName): An input to SIL is a device name (i.e., consumer friendly name or a unique ID). This is a use case of directly retrieving a TTM value previously measured and recorded by the same device elsewhere. The output TTM will be most accurate among the use cases.
2. SIL(DeviceProfile): An input to SIL is a device profile generated by the CDP. This is a use case when the same device information is not available in the lookup table. For example, a similarly configured device is located and its TTM is retrieved using the input device profile. Therefore, the output TTM is an estimated value.
3. SIL(BenchmarkScore): An input to SIL is a benchmark score. This is also a use case when the same device information is not available in the lookup table. For example, a device of comparable performance (i.e., in terms of benchmark score such as 100,000) is located and its TTM is retrieved. Consequently, the output TTM is also an estimated value.

**Algorithm 2** An algorithm for hierarchical side information lookup**Input:** A device profile $D_{i}$
**Output:** A TTM value ttm

 1:ttm←−1 /* initialization */ 2:**if** ($D_{i}$ exists in the lookup table) **then** 3:    $ttm\leftarrow SIL(D_{i})$ /* Case 1: *SIL(DeviceName) - Find TTM of the exact same device* */ 4:
**else**
 5:    $ttm\leftarrow SIL(D_{i})$ /* Case 2: *SIL(DeviceProfile) - Find TTM of similarly configured device* */ 6:    **if** (ttm==−1) **then** 7:        $ttm\leftarrow SIL(D_{i}._{side\_info})$ /* Case 3: *SIL(BenchmarkScore) - Find TTM of similar benchmark* */ 8:    **end if** 9:
**end if**
10:**return**ttm /* return retrieved or estimated TTM */


## 4. Experiments and Exemplar Cases

We evaluate our XDCC approach with eight Android smartphones and four smartwatches ranging from older low-end models to the latest high-end models, by a series of experiments and exemplar cases. These observations focus on the three procedures of the XDCC method including CDP (Section 4.1), TTM for on-device machine learning performance (Section 4.2) and exploiting AnTuTu and VFP benchmark scores in SIL (Section 4.3). Commercial Android-based smartphones and Wear OS smartwatches from Samsung, LG, Xiaomi, Sony and Motorola are used. For all experiments, we calculated average measurements while the highest value and the lowest value are both excluded.

### 4.1. Contextual Device Profiling for Smartphones and Smartwatches

**Figure 4:**
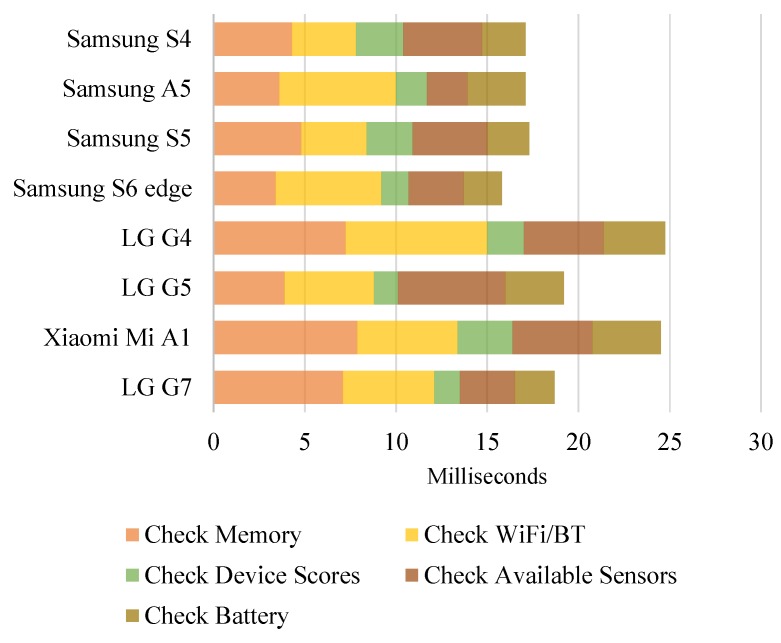
CDP on 8 commercial Android smartphones (lower is better).

**Figure 5:**
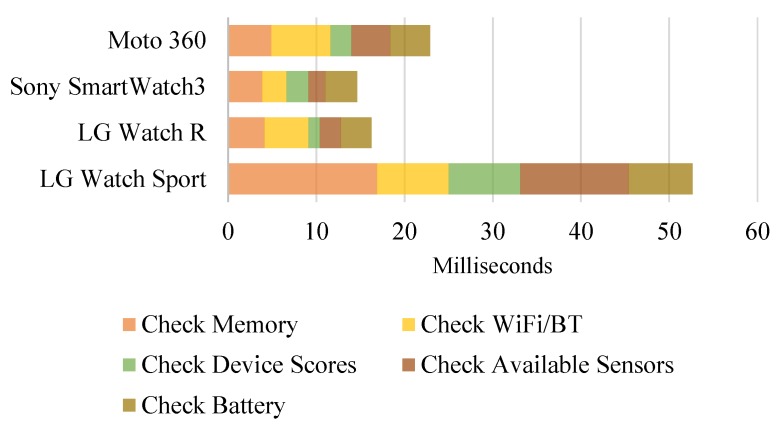
CDP on 4 Wear OS smartwatches (lower is better).

We developed and implemented a suite of CDP to obtain static and dynamic context (cf. Section 3.2) of the 12 devices. In our first experiment, we measured the time for our CDP method on various devices. We analyzed current statuses and usages of the devices in terms of memory, communication facilities, retrieving benchmark scores, sensors and battery. Figure 4 and Figure 5 show processing time required for running the suite of CDP on smartphones and smartwatches, respectively. On most devices, this CDP process took less than 25 ms (on 11 out of 12 devices). Implementation details for running this experiment using Android and Wear OS are presented.

- **Check Memory.** The total memory accessible by the kernel and available memory on the system are collected by accessing ActivityManager.MemoryInfo in Android API to determine current memory usage percentage and a low memory situation.
- **Check Wi-Fi and Bluetooth.** Current Wi-Fi network connection (i.e., connected or not and signal strength if connected) and Blutooth connection (i.e., on or off) statuses are reported by using ConnectivityManager and BluetoothAdapter of Android API.
- **Check Device Scores.** Previously measured AnTuTu and VFP benchmark scores are stored as a local database and a device model (Build.MODEL) is used as a key to retrieve associated benchmark scores as values.
- **Check Available Sensors.** Available sensors on the device are retrieved using SensorManager.
- **Check Battery.** Battery charging status, remaining battery percentage and battery temperature information are reported using BatteryManager.

### 4.2. Task Time Measurement for On-Device Machine Learning Performance

**Figure 6:**
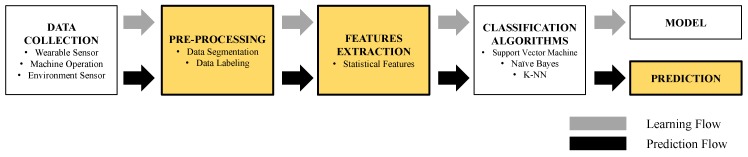
A typical machine learning flow. Highlighted processes are measured in TTM.

In our second experiment, we chose a relatively computation-intensive machine learning task to compare task performance on our target devices. Machine learning involves a pipeline of processes that is challenging for embedded systems such as mobile devices, wearables and IoTs due to the lack of computational power and resources. Figure 6 shows a typical machine learning flow. We evaluated and measured task time for completing a sequence of machine learning processes including preprocessing, feature extraction and model evaluation, on 12 target devices. Implementation details for running this experiment using Android and Wear OS are presented.

- **Preprocessing.** We have previously collected 9-axis sensor data from accelerometer, gyroscope, and linear acceleration and saved them as CSV (Comma Separated Value) files for machine learning applications. We used this dataset to evaluate preprocessing performance of reading, parsing and segmenting sensor data for a 1-second sliding window.
- **Feature Extraction.** As described in the work by [13] and [14], we used similar features to form two different feature sets for our experiment. First feature set includes a total of 84 features; 7 statistical features (mean, standard deviation, max, min, 3 quantiles) for 3 sensors’ 3 axis (x, y, z axis) and the magnitude (m) of the combined axes. Second feature set includes 84 features from the first set and additional 120 features from lower 10 bands produced by a Fast Fourier Transform (FFT) for 3 sensors’ x, y, z, and m.
- **Model Evaluation.** As a machine learning model, we used logistic regression classifier in scikit-learn scikit-learn, https://scikit-learn.org/) to train a model offline and deployed the trained model to Android and Wear OS devices in a form of PMML (Predictive Model Markup Language) (PMML, http://dmg.org/pmml/v4-3/GeneralStructure.html) for measuring model evaluation performance.

**Figure 7:**
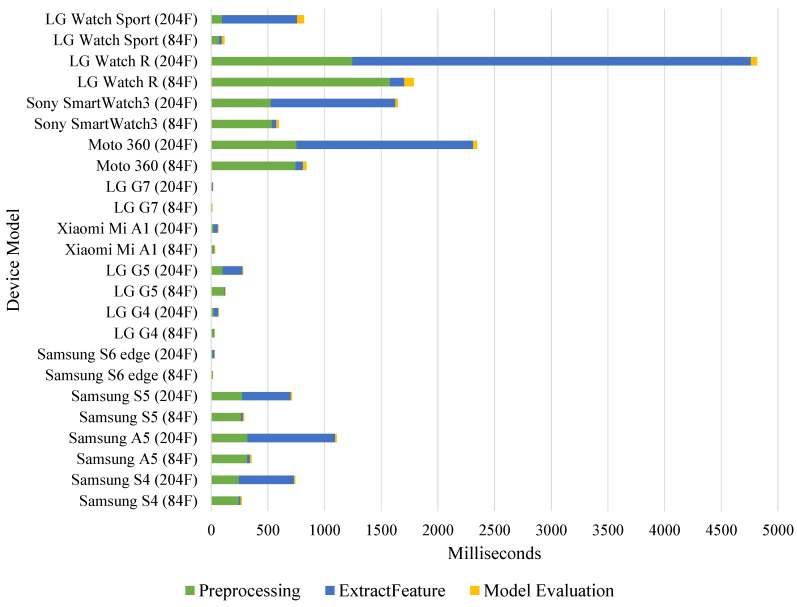
Processing time for 84 (84F) and 204 features (204F) extraction (lower is better).

Figure 7 shows TTM for preprocessing, feature extraction and model evaluation on Android smartphones and Wear OS smartwatches. When 84 features were extracted, all smartphones completed machine learning processes under 500 ms. However, when more demanding FFT features were included in 204 features, task time increased on all devices as shown in Figure 7.

#### 4.2.1. Effects of Low Battery

**Figure 8:**
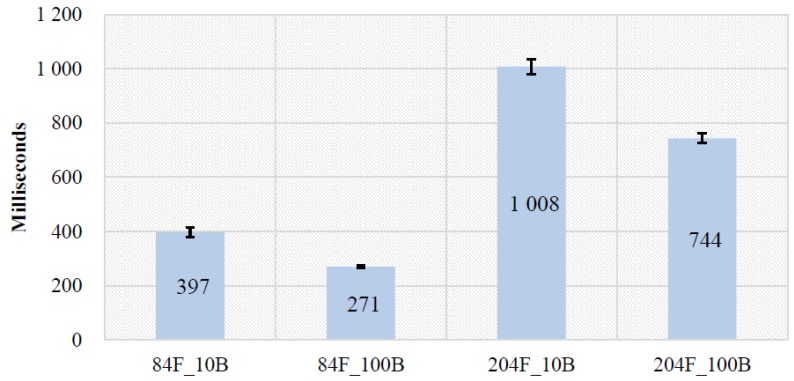
Effects of low battery on Samsung S4 (lower is better).

**Figure 9:**
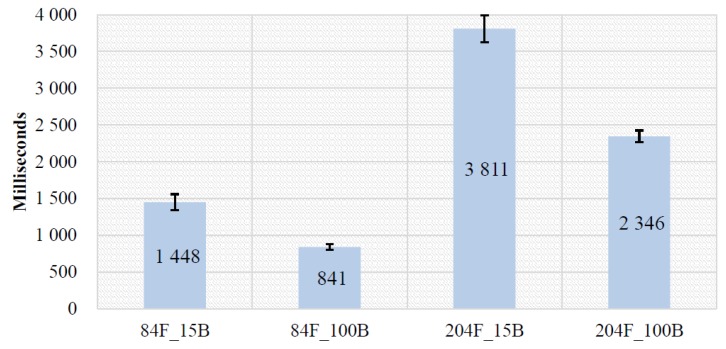
Effects of low battery on Moto 360 (lower is better).

We also examined effects of low battery in TTM to demonstrate that the sensed on-device context (i.e., battery status) can be used as a performance indicator for the XDCC problem. We used two devices that have low scores on AnTuTu and VFP benchmarks, including one smartphone (Samsung S4) and one smartwatch (Moto 360). The same task (cf. Section 4.2) is used for this experiment as well. For each device, fully charged (100% denoted as 100B) and low-battery (10–15% denoted as 10B and 15B) conditions were tested for two feature sets (84 features denoted as 84F and 204 features denoted as 204F). Figure 8 and Figure 9 show total processing time on Samsung S4 and Moto 360 for different battery conditions, respectively.

**Samsung S4.** A paired-samples t-test indicated that machine learning process time was significantly longer for 84F_10B (*M* = 397, *SD* = 24.9) than for 84F_100B (*M* = 271, *SD* = 8.02), *t*(7) = 14.6, *p* <0.001. The fully charged 84F_100B took, on average, 126 milliseconds less time than 84F_10B. Also machine learning process time was significantly longer for 204F_10B (*M* = 1008, *SD* = 39.0) than for 204F_100B (*M* = 743, *SD* = 25.5), *t*(7) = 12.2, *p* <0.001. The fully charged 204F_100B took, on average 265 milliseconds less time than 204F_10B.

**Moto 360.** A paired-samples t-test indicated that machine learning process time was significantly longer for 84F_15B (*M* = 1448, *SD* = 93.2) than for 84F_100B (*M* = 841, *SD* = 31.5), *t*(7) = 16.9, *p* <0.001. The fully charged 84F_100B took, on average, 607 milliseconds less time than 84F_15B. Also machine learning process time was significantly longer for 204F_15B (*M* = 3811, *SD* = 149) than for 204F_100B (*M* = 2346, *SD* = 63.4), *t*(7) = 22.3, *p* <0.001. The fully charged 204F_100B took, on average, 1465 milliseconds less time than 204F_15B.

### 4.3. AnTuTu and VFP Benchmark Scores as Side Information

As exemplar cases, we explored using AnTuTu and VFP benchmark scores as side information for SIL (cf. Section 3.4). AnTuTu Benchmark is the most used benchmarking app for Android devices. When a new Android smartphone is released, often their AnTuTu benchmark scores are measured and compared to previously released devices to show improvement. We downloaded AnTuTu benchmark app (AnTuTu Benchmark v7.0.8, https://play.google.com/store/apps/details?id=com.antutu.ABenchMark) from Google Play to run on Android devices. The benchmarking app was only used on smartphones, since there was no Wear OS specific version for smartwatches. Table 2 shows AnTuTu benchmark scores for all eight tested smartphones.

**Figure 10:**
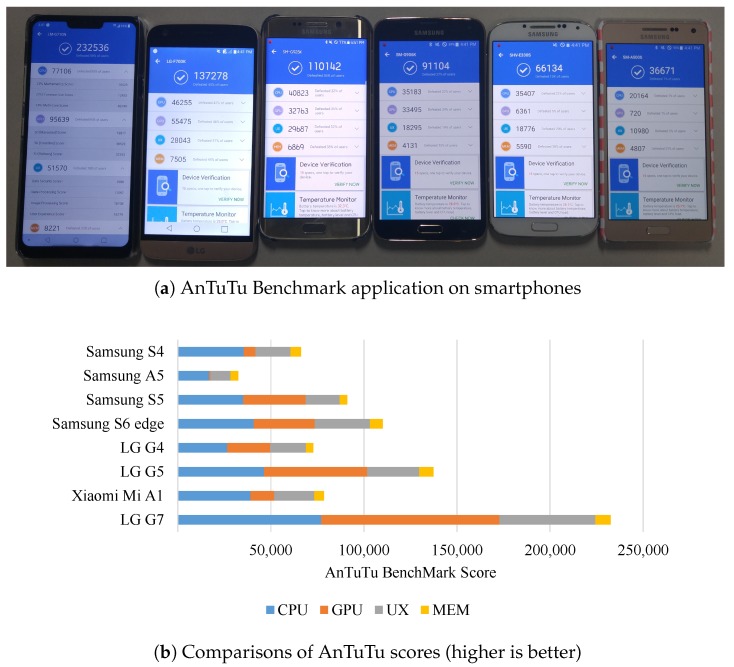
AnTuTu benchmark scores on Android smartphones.

This app runs various tests to measure and give separate scores on central processing unit (CPU), graphics processing unit (GPU), user experience (UX) and memory (MEM) aspects as shown in Figure 10a. As expected, the latest high-end smartphone (i.e., LG G7, which ranks 18th in Table 3) outperforms other devices in all aspects as shown in Figure 10b. We can leverage a publicly open lookup table (e.g., Table 3) to find similarly configured devices as well as similarly performing devices.

VFP Benchmark (https://dench.flatlib.jp/app/vfpbench) is another application to benchmark performance of floating point (FP) instruction set in GFLOPS (giga floating point operations per second) on Android and Wear OS. Specifically, this application tests single precision floating point, double precision floating point, single-thread and multi-thread. FP operations on embedded systems and IoTs are complex to perform than that of integers. Therefore, the measured GFLOPS of devices are suitable metrics to indicate their readiness for complex (i.e., FP) operation in terms of both processing time and precision. To obtain GFLOPS of target devices, we downloaded, installed, and ran Android (VFP Benchmark v1.3.4, https://play.google.com/store/apps/details?id=jp.flatlib.flatlib3.vfpbench) and Wear OS (VFP Benchmark for Android Wear v1.0, https://play.google.com/store/apps/details?id=jp.flatlib.flatlib3.vfpbenchw) versions of VFP Benchmark on 8 smartphones and 4 smartwatches, respectively.

**Figure 11:**
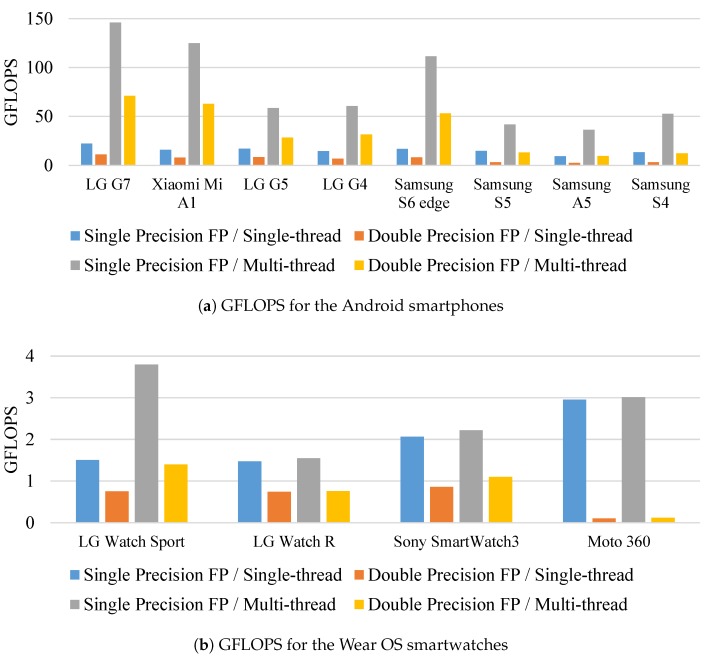
VFP benchmark results of Android devices (higher is better).

The results are summarized in Figure 11 and Table 4. As expected, FP operations on smartphones are 8 to 51 times faster than that of smartwatches. By using these side information (i.e., AnTuTu and VFP benchmarks) to build a lookup table and corresponding lookup functions in our XDCC method, we can directly retrieve benchmark scores, find devices with similar performance, find devices with comparable configuration and estimate performance of a new device without actually measuring task time.

### 4.4. Implemented Applications

To demonstrate feasibility of our approach, we present two applications (i.e., gesture recognition [14] and smart factory data collection [15]) on mobile collocated interactions with wearables from our earlier work.

**Figure 12:**
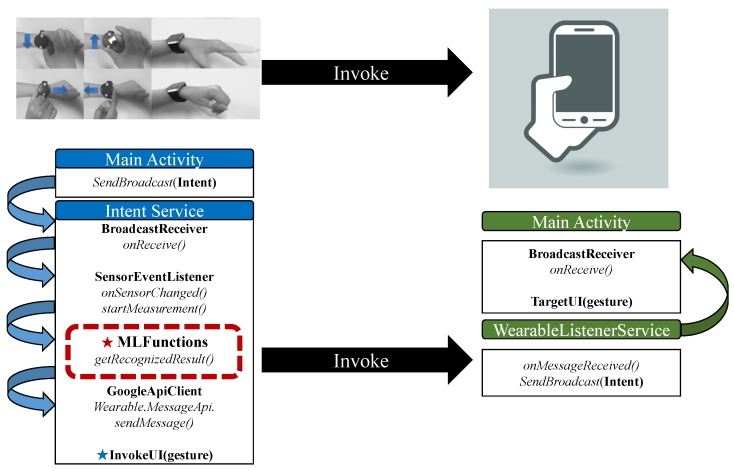
The motion UI application for MCI.

**Figure 13:**
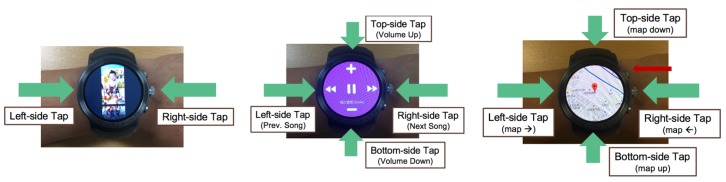
Motion UI-based gallery app, music player app and map app running on a smartwatch.

First gesture-recognition application uses the machine learning pipeline (cf. Section 4.2) to implement motion UI that recognizes the smartwatch wearer’s gestures for controlling gallery app, music player app and map app on the smartwatch [14]. As illustrated in our experiments and Figure 7, we implemented the motion UI with LG Watch Sport with 84 features. Figure 12 shows the motion UI application and its interfaces on a smartwatch for MCI. The machine learning pipeline denoted as MLFunctions is entirely run on the smartwatch and the recognized gesture is shared with the smartphone. Figure 13 shows the three implemented applications operated by the motion UI.

Second smart factory data collection application is implemented on less-powerful LG Watch R. As illustrated in our experiments, LG Watch R is not powerful enough to carry out heavy computation by itself. Therefore, the smartwatch is used to trigger starting and ending points of a task only while the collected data is stored on the smartwatch. The collected data is then transferred to a machine-environment local server at a later time.

Both applications show how different combinations of possible XDCC policies can be integrated at an application level. Different options on available devices (powerful vs. less-powerful wearables) and application demands (real-time gesture UI vs. data collection) should be carefully reviewed and reflected on implementing final applications.

## 5. Discussion

In this section, we discuss several implications and lessons learned for the XDCC problem based on our experiments results and exemplar cases.

First, a suite of contextual device profiling should be periodically or often checked before making computation coordination or offloading decisions. Since CDP running times for both smartphones (up to 25 ms) and smartwatches (up to 55 ms) are comparably modest, the accumulated time cost of checking would not be critical in most cases. At this stage, the device profile obtained from CDP should be used to eliminate mobile devices, wearables and IoTs that are physically incapable of executing XDCC processes further. To fully exploit device profiles, we can enrich these profiles with more complex details of the devices and make them publicly accessible for a certain group of tasks.

Second, as shown in the effects of low battery on machine learning processes (cf. Section 4.2.1), a device’s current battery status is important context to keep track of. The limitation of our experiment is that we only tested with smartphones and smartwatches. Since they have more flexibilities in terms of battery capacity than IoTs, performance degradation on IoTs with smaller battery capacities need to be further investigated. Nevertheless, battery usages of devices in MCI should be well managed in the XDCC method, so that the collaborative performance of the devices is significantly increased.

Third, previously measured benchmarking scores can be used to estimate performances of other mobile device, wearables and IoTs within similar ranges. For example, Samsung S4 and Samsung S5 have VFP scores within similar ranges, and their performance on machine learning processes also fall into the similar ranges (204 features, S4: 743 ms, S5: 713 ms and 84 features S4: 270 ms, S5: 291 ms). Similarly, we can infer that Samsung Note9 and Samsung S9 have comparable performance with LG G7, since they have similar AnTuTu scores shown in Table 3. So, if we only have AnTuTu or VFP scores, we can use benchmark scores as side information for backtracking and estimating performance on tasks of interest.

Lastly, if we want to deploy machine learning applications or any other computationally intensive task on mobile or wearable IoTs, features number and user-interaction time are important. Consider that our interactive application is required to process 2–3 inputs from users within a second. Based on our experiment results, using 204 features on any of the four smartwatches we tested, are not viable since their processing time are greater than 500 ms. Then we are forced to use only 84 features on LG Watch Sport or coordinate computation to smartphones.

## 6. Related Work

The proliferation of IoT has brought considerable changes in computing paradigms such as edge computing and fog computing. At the same time, poor computational capability and limited power capacity of current mobile and wearable IoTs pose problems on runtime performance degradation. Samie et al. identified four categories of IoT provided services and applications as one-to-one, one-to-many, many-to-one and many-to-many where IoT systems may exploit single/multiple devices to implement single/multiple applications [16]. For end-users to efficiently and effectively interact with the complicated IoT ecosystem, modern concepts such as cross-device interaction and offloading are becoming more relevant. In this section, we review related work on edge computing, fog computing, cross-device interaction and offloading, respectively. For more comprehensive and complete surveys, we direct readers to topical surveys for IoT technologies for embedded computing [16], mobile edge computing [17], fog computing [18,19], mobile multi-device ecosystems [5,7] and offloading [20,21].

### 6.1. Edge Computing

There is a branch of work focusing on roles and responsibilities of each individual device (i.e., mobile devices, wearables and IoTs). Lopez et al. proposed a vision of human-centered edge-device based computing where an edge may be a mobile device or a wearable device [22]. In their vision of “edge-centric computing”, edge devices of the network are proximate to end-users while having more control with support for mobile uses [22]. Shi and Dustdar argued that edge computing which refers to “the enabling technologies that allow computation to be performed at the network edge so that computing happens near data sources”, promotes many new IoT applications [23]. In edge computing, an edge can be “any computing and network resources along the path between data sources and cloud data centers [24]“. Shi et al. identified that edge computing has advantages in improving the total response time and energy consumption of devices by computing at the proximity of data sources [24]. Sun and Ansari proposed an IoT architecture edgeIoT [25] based on fog computing and software defined networking (SDN), to collect, classify and analyze the IoT data streams while minimzing the end-to-end delay between nodes. Satyanarayanan also discussed advantages of edge computing (i.e., the proximity of cloudlets) in four asepcts; these advantages included highly responsive cloud services, scalability via edge analytics, privacy-policy enforcement and masking cloud outages [26]. Mao et al. presented a survey on mobile edge computing (MEC) as a paradigm for distributing edges in order to provide “sufficient capacities for performing computation-intensive and latency-critical tasks at mobile devices [17]”. In their survey, MEC research themes across its components are categorized into computation task models, communication models, computation models of mobile devices and computation models of MEC servers, respectively [17]. Gu et al. proposed and formulated a task assignment problem between mobile edges as a one-to-many matching game, with the objective of minimizing the energy consumption [2].

Aligning our method within the theme of edge computing, our XDCC method aims to increase the overall performance of targets edge devices (i.e., mobile devices, wearables and IoTs) by executing XDCC tasks at the appropriate edge with physical capability and computation efficiency. Specifically, we investigated a particular MCI case where a user’s smartphone and smartwatch served the roles of edges.

### 6.2. Fog Computing

There is another branch of work that emphasize more on a distributed yet well-connected computing infrastructure. Dastjerdi and Buyya characterized fog computing as “a distributed paradigm that provides cloud-like services to the network edge” whose architecture consists of sensors and actuators employing “the sense-process-actuate and stream-processing programming models” [27]. Mukherjee et al. presented a survey to report on various architectures for fog computing and addressed open challenges of fog computing; some of the open research issues were application offloading, resource management and optimizing the reconfiguration cost in the SDN-based fog computing [18]. Sarkar et al. proposed a mathematical model to assess fog computing, in terms of power consumption, service latency, carbon dioxide emission and cost for high number of latency-sensitive applications [28]. Lavassani et al. proposed and demonstrated a model to save energy and reduce the number of packet transmissions in their fog computing testbed with IoT [3]. Bellavista et al. presented a conceptual architecture for Cloud-Fog-IoT applications that contains six different perspectives (1. communication, 2. security, 3. data quality, 4. sensing and actuaction management, 5. cloudification and 6. analytics and decision-making) [19].

In contrast to many studies that explore infrastructure-level issues in fog computing, our work focus on a task-level model and examines selected devices for achieving a mutual task effectively.

### 6.3. Cross-Device Interaction

XDI involves and poses many design, technological, social and perceptual challenges in mobile multi-device ecosystems [7,29]. Scharf et al. defined XDI as “the type of interaction, where human users interact with multiple separate input and output devices, where input devices will be used to manipulate content on output devices within a perceived interaction space with immediate and explicit feedback” [4]. In their work, four features (1. direct interaction with input devices, 2. mediated interaction, 3. perception of output devices, 4. immediate and explicit feedback) are used to systematically describe XDI [4]. Houben et al. used the term XDI more casually yet emphasized achieving a mutual task, “the seamless use of multiple devices to work toward achieving the same goal” [29]. Oh et al. proposed M+ as a platform-level solution to utilize application and system functionalities across Android devices [30]. Guo et al. built FoggyCache that demonstrates cross-device approximate computation reuse to minimize redundant computation in multi-devce mobile and edge scenarios [31]. Guo and Hu presented Potluck to achieve approximate deduplication in computation-intensive mobile applications [32].

Recently, there are several interactive scenarios for XDI in HCI research communities. Roels et al. presented INFEX that demonstrates a general framework for sharing information and UI on an interactive tabletop surface [33]. Serpi et al. proposed Web5VR as a framework for re-implementing manipulation techniques for different VR devices such as Kinect and Leap Motion [34]. For visualization on multi-devices, Langner et al. proposed VisTiles as a conceptual framework for visual data exploration specifically targeting co-located mobile devices [35]. For optimizing user interfaces in XDI, Park et al. proposed AdaM to optimize the allocation of UI elements in UI distribution [36].

In our work, we presented experiments and exemplar cases in MCI with wearables, which is a specific case of XDI. While XDI methods and applications presented in this section share the same motivation (i.e., achieving the same goal), our work focuses on a task-level XDCC with contextual device profiling and exploiting benchmark scores as usable side information.

### 6.4. Offloading

Computation or data offloading is a type of resource management actively pursued in edge computing, fog computing and XDI. Shi et al. proposed several guidelines of computational offloading for computationally demanding AR applications on wearable devices based on their experiences with AR applications on Google Glass [37]. Xu et al. identified several open challenges in algorithm design, incentive mechanism, user behavior utilization, security and privacy, computation-traffic offloading for realizing opportunistic offloading [20]. Hou and Xie proposed incentive mechanisms for mobile data offloading by considering social relationship of mobile users [38]. Ma et al. proposed the Computation Offloading Decision algorithm for IoT sensors where the offloading decision problem is formulated as a computation offloading game [1]. Ometov et al. proposed a mathematical model that delegates computing and caching functionality to resource-rich devices in the vicinity for AR applications scenarios [39]. Samie et al. proposed a computation offloading technique under bandwidth constraints to improve battery life of edge devices [40]. Chandra et al. summarized several approaches on offloading [21] to improve the battery life of mobile devices such as Somniloquy [41], WearDrive [42] and Mobile Assistance Using Infrastructure [43].

Compared to the related work, our XDCC method focuses on, (1) profiling cross-devices in MCI with wearables (i.e., smartphone + smartwatch) with benchmark scores as side information and (2) making an informed decision of offloading complex algorithms such as feature extraction in a typical machine learning application.

## 7. Conclusions

In this paper, we explored the concept of cross-device computation coordination in MCI and presented the XDCC method for performing XDCC concerning mobile devices, wearables and IoTs. A series of experiments and exemplar cases demonstrated that the XDCC method consists of CDP, TTM and SIL procedures provide insights for making computation offloading and XDCC decisions. Overall, the CDP procedure provided in the XDCC method is a light and fast module to be deployed onto both mobile and wearable IoTs for continuous monitoring the devices. Also exploiting side information such as AnTuTu and VFP benchmark scores as usable context, is practical and promising when some of the on-device context is missing or unavailable. As discussed in our exemplar cases, we can design and implement hierarchical SIL functions and lookup tables to retrieve or estimate a TTM value from similarly configured devices or similarly performing devices.

Our current approach is limited to computational tasks that involve floating point operations and machine learning tasks. Further studies are required for tasks that heavily use GPU (i.e., games and VR/AR applications) and network transfer (i.e., streaming applications). Moreover, since our work is not deployed to the real world setting to assess workload migration for specific tasks, this topic deserves further studies along with practical deployment and cross-device compatibility issues. Nevertheless, we expect that our proposed XDCC method to provide a means of harmoniously cooperating among mobile devices, wearables and IoTs while answering high application demands and conforming to IoT’s scalability, ubiquity and context-awareness requirements.

## Figures and Tables

**Table 1:** A suite of on-device static and dynamic context sensing for the device profiling.

Category	Static Context	Dynamic Context
Device Name	Consumer friendly name of a device	-
Screen	Screen size in pixels	-
Memory	Total memory accessible	Available memory
Battery Status	-	Charging/charged, battery level, temperature
Charging	-	USB charging, AC charging
Wi-Fi	Presence/Absence	Connected/disconnected, signal strength
Bluetooth	Presence/Absence	On/off
Sensors	Presence/Absence	Sensor values

**Table 2:** AnTuTu benchmark scores on the Android smartphones.

Device (Model No.)	Year	OS Ver.	CPU	GPU	UX	MEM	Total Score
LG G7 (LM-G710N)	2018	8.0	77,106	95,639	51,570	8221	232,536
Xiaomi Mi A1 (Mi A1)	2017	8.0	39,127	12,700	21,540	5215	78,582
LG G5 (LG-F700K)	2016	6.0	46,255	55,475	28,043	7505	137,278
LG G4 (LG-F500K)	2015	7.0	26,637	23,055	19,161	4016	72,869
Samsung Galaxy S6 edge (SM-G925K)	2015	6.0	40,823	32,763	29,687	6869	110,142
Samsung Galaxy S5 (SM-G906K)	2014	6.0	35,183	33,495	18,295	4131	91,104
Samsung Galaxy A5 (SM-A500S)	2014	6.0	20,164	720	10,980	4807	36,671
Samsung Galaxy S4 (SHV-E330S)	2013	5.0	35,407	6361	18,776	5590	66,134

**Table 3:** Top 50 AnTuTu benchmark scores on popular Android smartphones (as of November 2018), retrieved from http://www.antutu.com/en/ranking/rank6.htm.

Device Ranking	RAM+Storage	CPU	GPU	3D	Total Score
1. OnePlus 6T	8GB+128GB	92,504	64,447	127,682	295,181
2. Mi MIX 2S	6GB+64GB	89,139	61,097	127,509	287,111
3. OnePlus 6	8GB+256GB	91,547	63,968	127,359	293,745
4. Mi Black Shark	8GB+128GB	91,747	61,101	126,599	291,099
5. ROG Phone	8GB+512GB	94,170	63,449	126,517	297,019
6. Mi 8	6GB+256GB	91,168	59,745	125,970	287,142
7. vivo NEX S	8GB+128GB	92,080	57,043	125,929	286,433
8. Samsung Note9 (SDM845)	6GB+128GB	89,058	59,787	125,893	283,004
9. Mi POCOPHONE F1	6GB+128GB	89,082	59,735	125,786	283,861
10. Meizu 16th	8GB+128GB	92,110	60,562	123,425	286,943
11. Sony Xperia XZ2	4GB+64GB	84,402	61,151	120,998	275,832
12. HUAWEI Mate 20 Pro	6GB+128GB	112,070	68,221	110,574	305,437
13. HUAWEI Mate 20	6GB+128GB	111,964	68,069	110,195	304,306
14. HUAWEI Mate 20 X	6GB+128GB	111,156	67,550	109,787	301,661
15. Samsung S9+ (SDM845)	6GB+64GB	89,216	58,474	108,415	264,543
16. ZenFone 5Z	8GB+256GB	90,665	59,079	106,785	268,858
17. Samsung S9 (SDM845)	4GB+64GB	89,271	58,485	106,389	262,421
18. LG G7 ThinQ	4GB+64GB	87,647	57,084	104,412	257,715
19. Samsung Note9 (9810)	6GB+128GB	85,108	53,597	96,578	243,362
20. Samsung S9+ (9810)	6GB+64GB	89,626	55,646	94,284	247,968
21. Samsung S9 (9810)	4GB+64GB	89,406	55,602	92,800	246,188
22. Google Pixel 2 XL	4GB+128GB	71,089	43,540	90,138	213,603
23. Moto Z2 Force	6GB+64GB	72,094	44,244	83,147	207,589
24. Samsung Note8 (SDM835)	6GB+64GB	68,902	44,700	82,269	203,128
25. Nokia 8	4GB+64GB	72,197	45,851	81,928	208,422
26. HONOR V10	6GB+128GB	69,932	44,528	80,697	208,670
27. HUAWEI Mate 10 Pro	6GB+128GB	71,013	44,408	80,037	209,042
28. Samsung Note 8 (8895)	6GB+64GB	69,323	44,001	79,582	200,533
29. HUAWEI P20 Pro	6GB+128GB	71,799	46,324	78,184	209,863
30. HONOR 10	6GB+128GB	68,930	46,318	78,041	206,674
31. HUAWEI P20	4GB+128GB	71,706	45,804	77,832	208,795
32. Samsung S8 (SDM835)	4GB+64GB	68,472	43,989	77,791	197,129
33. LG V30	4GB+128GB	57,810	33,100	77,698	175,130
34. HONOR Play	4GB+64GB	70,923	45,702	77,379	207,310
35. Samsung S8 (8895)	4GB+64GB	67,549	43,731	77,173	195,700
36. Samsung S8+ (SDM835)	4GB+64GB	68,594	43,853	77,015	197,071
37. Samsung S8+ (8895)	4GB+64GB	62,296	43,192	76,839	189,122
38. HUAWEI nove 3	4GB+128GB	70,499	45,594	76,712	206,140
39. Essential Phone	4GB+128GB	70,083	44,482	73,577	197,362
40. LG G5	4GB+32GB	46,147	35,129	63,673	150,769
41. LG G6	4GB+32GB	51,643	36,278	60,847	153,761
42. Mi 8 SE	4GB+64GB	66,640	44,511	47,843	168,135
43. Mi 8 Lite	4GB+64GB	66,866	38,965	30,409	143,922
44. Nokia 7 Plus	4GB+64GB	64,629	38,908	30,355	140,502
45. Mi 6X	6GB+64GB	62,818	37,222	30,153	138,238
46. vivo V9	6GB+64GB	62,404	38,079	30,139	138,050
47. OPPO F7 Youth	4GB+64GB	62,427	36,331	29,668	137,936
48. HONOR 8X	4GB+64GB	66,921	37,717	22,571	139,794
49. HUAWEI Mate 20 Lite	4GB+64GB	66,201	37,619	22,566	138,890
50. HUAWEI nova 3i	4GB+64GB	66,116	37,614	22,489	138,671

**Table 4:** VFP benchmark results of floating point (FP) instruction sets on the Android and Wear OS devices (SP: Single Precision, DP: Double Precision, S-Thread: Single-thread, M-Thread: Multi-thread).

Device	SP S-Thread	DP S-Thread	SP M-Thread	DP M-Thread	ARCH	CPU Core
LG G7	22.237	11.122	145.945	71.15	ARMv8A	8
Xiaomi Mi A1	15.774	7.883	124.874	62.88	ARMv8A	8
LG G5	17.131	8.484	58.55	28.339	ARMv8A	4
LG G4	14.522	6.739	60.698	31.537	ARMv8A	6
Samsung S6 edge	16.721	8.382	111.476	53.152	ARMv8A	8
Samsung S5	14.634	3.27	41.851	13.215	ARMv7A	4
Samsung A5	9.32	2.512	36.326	9.68	ARMv7A	4
Samsung S4	13.454	3.238	52.716	12.306	ARMv7A	4
LG Watch Sport	1.508	0.755	3.796	1.398	ARMv7A	4
LG Watch R	1.476	0.739	1.545	0.757	ARMv7A	4
Sony SmartWatch3	2.061	0.862	2.216	1.097	ARMv7A	4
Moto 360	2.955	0.105	3.016	0.117	ARMv7A	1

## Abbreviations

The following abbreviations are used in this manuscript:

AR	Augmented Reality
CDP	Contextual Device Profiling
CSV	Comma Separate Value
DP	Double Precision
FFT	Fast Fourier Transform
GFLOPS	Giga FLoating point OPerations per Second
GPU	Graphical Processing Unit
HCI	Human-Computer Interaction
IoT	Internet-of-Things
MCI	Mobile Collocated Interactions
MEM	Memory
PMML	Predictive Model Markup Language
SDN	Software Defined Network
SIL	Side Information Lookup
SP	Single Precision
TTM	Task Time Measurement
UI	User Interface
UX	User Experience
VR	Virtual Reality
XDCC	Cross-Device Computation Coordination
XDI	Cross-Device Interaction
